# Clinical and economic burden of invasive meningococcal disease: Evidence from a large German claims database

**DOI:** 10.1371/journal.pone.0228020

**Published:** 2020-01-28

**Authors:** Liping Huang, Olivia Denise Heuer, Sabrina Janßen, Dennis Häckl, Niklas Schmedt

**Affiliations:** 1 Pfizer Inc., Collegeville, PA, United States of America; 2 Institute for Applied Health Research Berlin, Berlin, Germany; 3 Pfizer Deutschland GmbH, Berlin, Germany; 4 WIG2 GmbH, Leipzig, Germany; Public Health England, UNITED KINGDOM

## Abstract

**Background:**

Limited data is available to describe clinical characteristics, long-term outcomes, healthcare resource use and the attributable costs of invasive meningococcal disease (IMD) in Germany. We aimed to examine demographic and clinical characteristics as well as healthcare resource use and related costs.

**Methods:**

We conducted a retrospective cohort study based on the InGef database in patients with IMD between 2009 and 2015. Cases were identified based on hospital main discharge diagnoses of IMD. Demographics, clinical characteristics, 30-day and 1-year mortality as well as IMD-related complications and sequelae in IMD cases were examined. In addition, short and long-term costs and healthcare resource use in IMD cases were analyzed and compared to an age- and sex-matched control group without IMD.

**Results:**

The study population comprised 164 IMD cases between 2009 and 2015. The mean length of the IMD-related hospitalization was 13 days and 38% of all cases presented with meningitis only, 35% with sepsis only, 16% with both and 11% with other IMD. The 30-day and one-year mortality were 4.3% and 5.5%, respectively. Approximately 13% of IMD cases had documented IMD-related complications at hospital discharge and 24% suffered from sequelae during follow-up. The IMD-related hospitalization was associated with mean costs of € 9,620 (standard deviation: € 22,197). The difference of mean costs between IMD cases and matched non-IMD controls were € 267 in the first month and € 1,161 from one month to one year after discharged from IMD-related hospitalization. During the later follow-up period, the mean overall costs and costs associated with individual healthcare sectors were also higher for IMD cases without reaching statistical significance.

**Conclusions:**

IMD resulted in severe complications and sequelae and was associated with extensive costs and increased healthcare resource use in Germany, especially in the first year after IMD diagnosis and due the IMD-related hospitalization.

## Background

Invasive meningococcal disease (IMD) is caused by invasion of *Neisseria meningitidis (N*. *meningitides)* into the blood stream and/or central nervous system resulting in meningitis, septicemia as well as less frequent manifestations such as arthritis and pericarditis. Clinical symptoms are often nonspecific including headache and fever and develop in the course of an infection into more specific symptoms for meningitis or sepsis like neck stiffness, hemorrhagic rash (pupura fulminans) as well as altered consciousness and lethargy in advanced disease [1]. In general, twelve different serogroups of *N*. *meningitidis* exist, but the majority of IMD cases are caused by serogroups A, B, C, X, Y, and W [2].

Despite availability of antibiotic treatment, IMD remains a serious public health concern. The incidence in Europe and the United States was less than one case per 100,000 persons in 2016 [3,4] compared to the incidence in the epidemic regions in sub-Saharan Africa with incidence of 10 to 1,000 cases per 100,000 population [5]. Typically, the highest incidence is observed in infants and young children followed by a smaller peak in adolescents and young adults [2,5]. In Germany, IMD is classified as notifiable disease and must be reported to local health authorities by physicians and laboratories according to a standardized case definition [6]. A total number of 338 IMD cases were reported according to the infectious disease surveillance in 2016 with the highest incidence observed in children aged 0 to 4 years and a second peak in adolescents aged 15 to 19 years. The case fatality rate of IMD is high with 5–15% and up to 57% of survivors in adolescents aged 15 to 19 years develop a wide range of sequelae [7–11] such as hearing loss, visual impairment, neurological impairments or limb amputation. As a result, IMD is associated with substantial short-term and long-term costs for health care systems [12–14].

In Germany, limited data is available on detailed clinical characteristics, long-term outcomes, health care resource use or the attributable costs of IMD, although IMD is part of the infectious disease surveillance. Therefore, we aimed to examine demographic and clinical characteristics as well as healthcare resource use (HRU) and related costs in Germany.

## Methods

### Data source

This study was based on claims data from the InGef (Institute for Applied Health Research Berlin) (former Health Risk Institute) database which includes longitudinal inpatient and outpatient claims data of statutory health insurance providers (SHIs) in Germany [15]. For this study, we used data of approximately 8 million insured members of 61 SHIs covering approximately 9.7% of the German population. In Germany, health insurance is mandatory and the majority of the population (87.9%) is insured in one of 110 public SHIs (as of 2018) [16].

In brief, the InGef database includes demographic data; ambulatory services and diagnoses; hospital data including admission and discharge dates, the main and secondary discharge diagnoses and procedures performed in hospital; drug prescription and dispensing data; reimbursed remedies (e.g. physical or occupational therapy) and aids; and costs of each healthcare sector, i.e. ambulatory services, hospitalization, drugs, remedies and aids, from the perspective of the German SHIs. All diagnoses in the InGef database are coded according the German modification of the 10th revision of the International Classification of Diseases (ICD-10 GM) [17].

Data contributing to the InGef database are stored at a specialized data center according to §284 in combination with §70 and §71 Social Code Book (“Sozialgesetzbuch”, SGB) V [18]. The data center is owned by SHIs and provides data warehouse services. In the data center (acting as a trust center), data with respect to individual insured members and health care providers (e.g. physicians, practices, hospitals, pharmacies) are anonymized by coarsening or by removing individual variables. Since all patient-level data in the InGef database are no longer social data according to § 67 Abs. 2 SGB X [19] in combination with Art. 4 Nr. 1 of the General Data Protection Legislation (“Datenschutz-Grundverordnung”, DSGVO) [20], institutional review board/ethical approval and informed consent of the patient was not required.

### Study design and setting

A retrospective cohort study was conducted based on IMD cases identified from the InGef database. Patients were eligible to enter the study if they had at least one hospitalization with IMD (ICD-10 GM code A39) as main discharge diagnosis between 2009 and 2015 (enrollment period) and valid information on age and sex. In addition, in order to ensure IMD cases included in the study were newly diagnosed cases, patients must not have any hospital or ambulatory diagnosis of IMD one year prior to or from birth onwards to the admission date of the first IMD-related hospitalization (baseline period). The first hospitalization due to IMD during the enrollment period was defined as index hospitalization. Patients with IMD were further classified into the following mutually exclusive groups under consideration of secondary hospital discharge diagnoses: Meningitis (ICD-10 GM A39.0) + sepsis (ICD-10 GM A39.2, A39.3, A39.4), meningococcal meningitis only (ICD-10 GM A39.0), meningococcal sepsis only (ICD-10 GM A39.2, A39.3, A39.4) and other IMD including Waterhouse-Friderichsen syndrome, meningococcal heart disease, other meningococcal infections and unspecified meningococcal disease (ICD-10 GM A39.1, A39.5, A39.8, A39.9).

For the purpose of comparing HRU and associated costs in IMD cases with the general SHI population in Germany, a case-matched cohort study using an age and sex-matched control group was selected. Matching by age and sex was performed to balance HRU and costs between IMD cases and controls to allow for a fair comparison of HRU and costs during follow-up. Patients without any diagnosis of IMD from the database were eligible to be included as potential controls, if they had at least one year of continuous insurance before the cohort entry date or from birth onwards to the cohort entry date (baseline period). The cohort entry date for potential controls was assigned to the beginning of the quarter of the respective IMD case to ensure that the distribution of the cohort entry dates of the controls is similar to the distribution of the cohort entry dates of IMD cases. For each IMD case, up to four controls were matched without replacement in the respective calendar quarter by age and sex (1:4 matching). All IMD cases and matched controls without IMD were followed up from the discharge date of index hospitalization or the cohort entry date, respectively, for a maximum of seven years until 31 December 2016 (end of the study period), disenrollment of SHI or death, whichever occurred first (follow-up period).

### Statistical analysis

The distribution of IMD cases by age group, sex and clinical presentation, as well as presence of immunodeficiencies considered as risk factors according to the German Standing Committee on Vaccination (STIKO, “Ständige Impfkomission”) assessed in the baseline period, mortality, presence of IMD-related complications at index hospital discharge and during the entire study period, and the presence of sequelae were descriptively examined. The presence of IMD-related complications was assessed based on diagnoses Waterhouse-Friedrichsen syndrome (which may imply adrenal hemorrhage), anoxic brain damage, or stroke. The presence of sequelae was defined as the presence of limb ataxia, limb amputation, paresis, paralysis, obstructive hydrocephalus, cranial nerve palsy, learning disabilities, mental retardation, blindness, hearing loss, skin necrosis and/or skin grafting, chronic renal failure, or epilepsy and seizures during the study period. All underlying definitions associated with risk factors, complications, and sequelae are displayed in S1 Appendix.

The overall 30-day and the one-year mortalities were calculated as the proportion of IMD cases who died within 30 days or within one-year after index hospital admission date, respectively, relative to the total number of patients in the IMD cohort. The date of death was assessed as date of SHI disenrollment with death as the documented reason for disenrollment. The corresponding 95%-confidence intervals were calculated assuming a binomial distribution.

The overall costs and the costs associated with individual healthcare sectors, i.e. ambulatory care, hospitalization, drug utilization, and remedies and aids were descriptively examined. Based on data of the “Organisation for Economic Co-operation and Development” [21], all costs, including costs during the index hospitalization and during the follow-up period were standardized to the year 2016 to account for inflation over time.

Two multivariable analyses were conducted to examine HRU measured by length of stay of the index hospitalization (LOS) and costs associated with the index hospitalization for the IMD cohort. A multivariable negative binominal regression was used to estimate adjusted ratio of LOS between a category of interest versus a category of reference of covariates, which included age group (1 year, 1–4 years, 5–9 years, 10–17 years, 18–24 years, 25+ years), sex (male, female), clinical presentation of IMD (meningitis + sepsis, meningitis only, sepsis only, other IMD), presence of risk conditions according to STIKO (yes, no) and presence of IMD-related complications at discharge of the index hospitalization (yes, no). Costs associated with the index hospitalization were analyzed using a multivariable gamma regression model. Ratios of total cost per day between a category of interest and a reference group were reported, adjusting for the same covariates included in the multivariable negative binominal regression for LOS.

For the analysis of short-term and long-term costs in IMD cases, we stratified the follow-up period into the following periods to account for different length of study follow-up time: < = 1 month, 1 month to 1 year, 1 year to 3 years, 3 years to 5 years and 5 to 7 years. We considered <1 month and 1 month to 1 year as a short-term follow-up period and later time periods as a long-term follow-up period. A descriptive analysis was performed to examine mean overall costs during the index hospitalization and during the different follow-up periods. A two-part regression model was applied to estimate adjusted ratios of total cost per day during the follow-up period between a category of interest and a reference group of covariates, including age group (1 year, 1–4 years, 5–9 years, 10–17 years, 18–24 years, 25+ years), sex (male, female), clinical presentation of IMD (meningitis + sepsis, meningitis only, sepsis only, other IMD), presence of risk conditions (yes, no) and presence of IMD-related complications at discharge of the index hospitalization (yes, no), presence of IMD-related complications during the follow-up period (yes, no), and presence of IMD-related sequelae during the follow-up period (yes, no). The two-part model was composed of a logistic regression model in all IMD cases with an indicator of non-zero costs as a dependent variable, and a gamma regression model in IMD cases with costs greater than zero and the total cost per day as a dependent variable.

HRU and associated costs during the follow-up period for IMD cases with at least one day of follow-up were compared to the matched controls without IMD. Negative binominal regression was applied to estimate HRU rate ratios on the number of hospitalizations, number of ambulatory physician visits and number of different drugs used in relation to IMD status (yes, no) for the overall follow-up period up to seven years as well as for the follow-up periods of < = 1 month, 1 month to 1 year, 1 year to 3 years, 3 years to 5 years and 5 to 7 years. Further, a two-part bootstrapping regression was applied to estimate rate ratios of overall cost per day as well as costs per day for individual healthcare sectors as along with bootstrap 95% confidence intervals (CI) and p-values for the follow-time periods depending on IMD status (yes, no). Again, the two-part model was composed of a logistic regression model in all individuals with an indicator of non-zero costs as a dependent variable, and a gamma regression model in individuals with costs greater than zero and the cost per day as a dependent variable.

All analyses were conducted using the Statistical Analysis System (SAS) statistical software package, version 9.4.

## Results

The study population comprised 164 IMD cases between 2009 and 2015 selected from a source population of approximately 8 million insured members of SHIs (S2 Appendix).

### Demographic and clinical characteristics of IMD cases

Demographic and clinical characteristics of all IMD cases are displayed in Table 1. Mean age at diagnosis was 24.2 years (standard deviation (SD) 23.6 years) and 51% were female. One-fifth of cases occurred in the age group 1 to 4 years (20.1%). More than 30% of IMD cases occurred in adolescents and young adults aged 10 to 24 years of age, whereas 34.8% of the IMD cases affected persons aged 25 years or older. Of the total number of IMD patients, 12.8% had a risk factor according to STIKO’s definition.

**Table 1:** Demographic and clinical characteristics of IMD patients between 2009 and 2015 in Germany.

	N IMD cases or Mean	% or SD
**Sex**		
Men	81	49.4
Women	83	50.6
**Age at IMD diagnosis**		
Age (Mean and SD)	24.2	23.6
<1 year^a^	16	9.8
0 - <3 months	N < 5	
3 - <6 months	7	4.3
6 - <9 months	5	3.1
9 - <12 months	N < 5	
1–4 years	33	20.1
5–9 years	6	3.7
10–17 years	22	13.4
18–24 years	30	18.3
25+ years	57	34.8
**Presence of risk factor according to STIKO**^**b**^	21	12.8
**Clinical presentation of IMD**		
Meningitis + sepsis	26	15.9
Meningitis only	62	37.8
Sepsis only	58	35.4
Other IMD^c^	18	11
**Mortality**		
30-day mortality	7	4.3 (95%-CI: 1.7–8.6)
1-year mortality	9	5.5 (95%-CI: 2.5–10.2)
**Presence of IMD complication at index hospital discharge**^**d**^	21	12.8
**Presence of IMD complication during the study period**^**d**^	24	14.6
Waterhouse-Friderichsen syndrome covering adrenal hemorrhage	19	11.6
Anoxic brain damage	N <5	
Stroke	6	3.7
**Presence of Sequelae occurred during the study period**^**e**^	35	23.5
Limb ataxia, paresis, and paralysis (0 patients excluded)	9	5.5
Cranial nerve palsy (1 patients excluded)	N <5	
Learning disabilities and mental retardation (3 patients excluded)	N <5	
Blindness (0 patients excluded)	N <5	
Obstructive hydrocephalus (0 patients excluded)	N <5	
Hearing loss (5 patients excluded)	9	5.7
Skin necrosis and/or skin grafting (1 patients excluded)	6	3.7
Limb amputation (0 patients excluded)	N <5	
Chronic renal failure (3 patients excluded)	12	7.5
Epilepsy and seizures (3 patients excluded)	11	6.8

IMD = Invasive meningococcal disease; STIKO = Standing Committee on Vaccination (Germany); Information for patient groups of 1 to less than 5 are not displayed due to data protection reasons, zeros are shown

a n = 12 (75%) of IMD patients in children <1 year occurred in month 3–9

b Include functional or congenital asplenia, defects of the complement system, immunodeficiency with predominantly antibody defects, combined immunodeficiency, immunodeficiency associated with other major defects, common variable immunodeficiency, neutropenia and functional disorders of polymorphonuclear neutrophils, transplanted organ and tissue status and cochlear implant, malignant neoplasm, radiation therapy, HIV infection, and other hematological disease sickle-cell disorders

c Include Waterhouse-Friderichsen syndrome covering adrenal hemorrhage, meningococcal heart disease, other meningococcal infections and unspecified meningococcal disease

d Include Waterhouse-Friderichsen syndrome covering adrenal hemorrhage, anoxic brain damage, stroke

e Include limb ataxia, paresis, and paralysis, obstructive hydrocephalus, cranial nerve palsy, learning disabilities and mental retardation, blindness, hearing loss, skin necrosis and/or skin grafting, limb amputation, chronic renal failure, and epilepsy and seizures; patients with a prior diagnosis of a sequelae in the baseline period were excluded

Considering the clinical presentation of IMD, most of patients were hospitalized with presentation of meningitis only (37.8%) followed by sepsis only (35.8%). Clinical presentation of meningitis plus sepsis was descriptively less frequent (15.9%). The 30-day and 1-year mortalities were 4.3% (95% CI: 1.7%-8.6%) and 5.5% (95% CI: 2.5%-10.2%), respectively. Of all IMD cases, 12.8% had at least one documented IMD-related complication at discharge of the index hospitalization and 23.5% were diagnosed with at least one sequelae during the follow-up period. The most frequent IMD-related complication and sequelae were Waterhouse-Friderichsen syndrome covering adrenal hemorrhage (11.6%) and chronic renal failure (7.5%), respectively. Demographic and clinical characteristics of IMD cases stratified by age group are shown in S2 Appendix.

### Healthcare resource use and attributable costs

**Fig 1:**
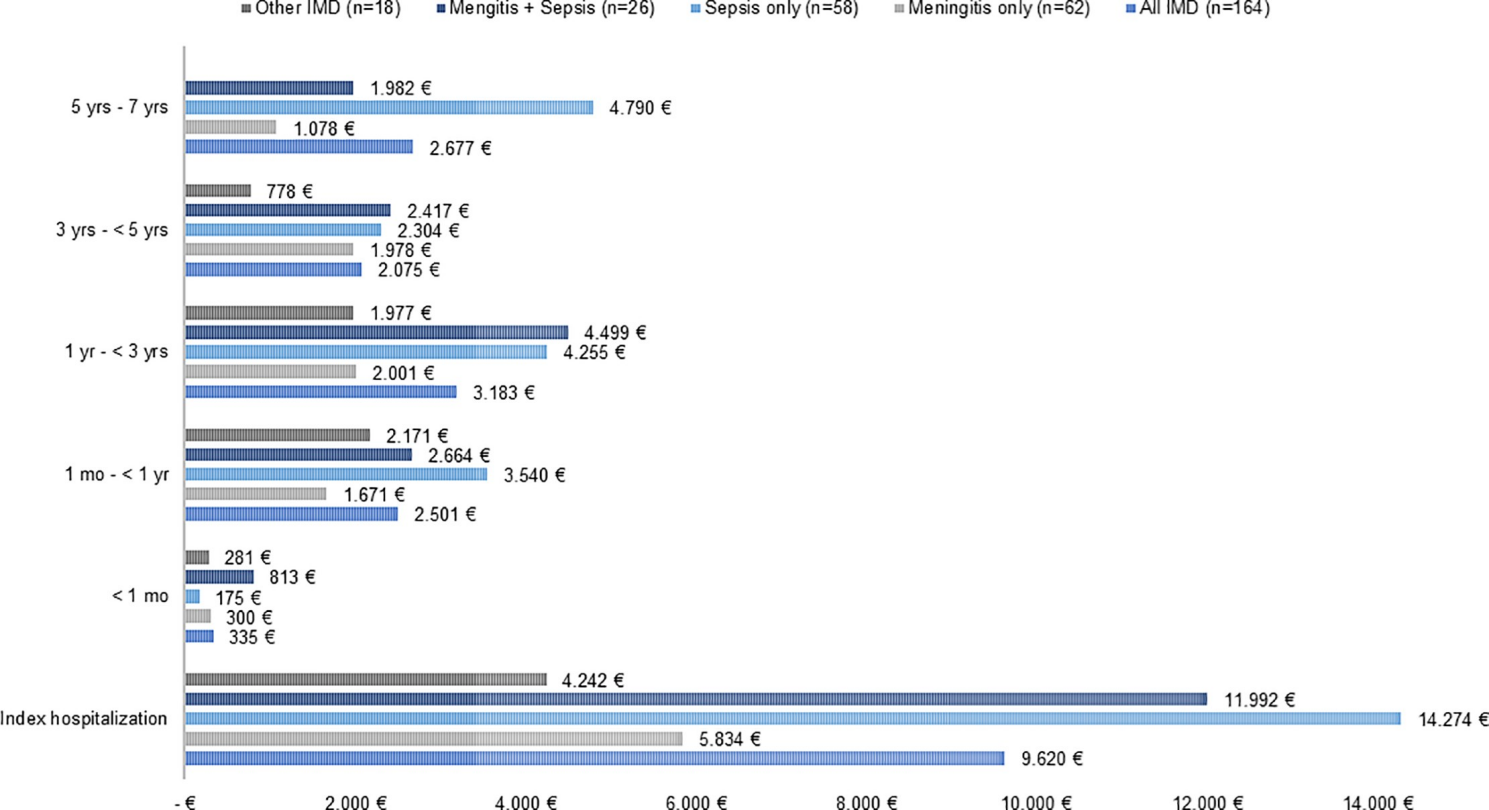
Mean overall costs in Euro (€) of IMD cases within pre-defined follow-up periods after diagnosis and stratified by clinical presentation of IMD. IMD = Invasive meningococcal disease; Data for other IMD in the time period 5 to 7 years could not be displayed due to data protection reasons (n<5); further information on SD and costs by clinical presentation of IMD in each healthcare sector is available in S2 Appendix.

Descriptively, the mean overall costs during the index hospitalization and during different follow-up periods after discharge from index hospitalization in all IMD patients stratified by clinical presentation is presented in Fig 1. The costs of the index hospitalization as well as costs in different follow-up periods were slightly higher in patients with meningitis plus sepsis and sepsis only compared to those with meningitis only or those with other type of IMD except the first month after discharge from index hospitalization.

### IMD-related HRU and costs

The mean and median LOS of the IMD-related index hospitalization were 13.1 days (SD 11.3 days) and 11 days (Q1 8 days; Q3 15 days), respectively (data not shown). The result from the multivariable analysis showed that, compared with the infant group (i.e., aged < 1 year), LOS did not differ across age groups except for the age group 5 to 9 years, in which the LOS was 50% of the infant group (Table 2, adjusted LOS ratio: 0.50; 95% CI: 0.26–0.96). Similarly, LOS of IMD cases presenting as other type of IMD was only 38% of LOS of patients with meningitis only (adjusted LOS ratio: 0.38; 95% CI: 0.26–0.56). IMD cases with presence of IMD-related complications at discharge of the index hospitalization stayed 150% longer than those without complications (adjusted LOS ratio: 1.51; 95% CI: 1.08–2.11).

**Table 2:** Adjusted ratio of length of stay of IMD-related index hospitalization (2009–2015).

	LOS ratio^a^ (95%-CI)	p-value^a^
**Age (ref. <1 year)**		
1–4 years	0.97 (0.65–1.44)	0.868
5–9 years	0.50 (0.26–0.96)	0.037
10–17 years	0.95 (0.62–1.46)	0.820
18–24 years	1.00 (0.66–1.52)	0.988
> = 25 years	1.32 (0.90–1.93)	0.150
**Sex (ref. females)**	1.10 (0.89–1.37)	0.384
**Clinical presentation of IMD (ref. meningitis only)**		
Meningitis + sepsis	0.96 (0.71–1.30)	0.788
Sepsis only	0.88 (0.68–1.13)	0.321
Other IMD^b^	0.38 (0.26–0.56)	<0.001
**Presence of risk factor according to STIKO**^**c**^ **(ref. no)**^**c**^	0.84 (0.64–1.11)	0.231
**Presence of IMD related complication at hospital discharge (ref. no)**^**d**^	1.51 (1.08–2.11)	0.017

CI = Confidence interval, IMD = Invasive meningococcal disease; STIKO = Standing Committee on Vaccination (Germany)

a obtained from multivariable negative-binomial regression model

b Include Waterhouse-Friderichsen syndrome covering adrenal hemorrhage, meningococcal heart disease, other meningococcal infections and unspecified meningococcal disease

c Include functional or congenital asplenia, defects of the complement system, immunodeficiency with predominantly antibody defects, combined immunodeficiency, immunodeficiency associated with other major defects, common variable immunodeficiency, neutropenia and functional disorders of polymorphonuclear neutrophils, transplanted organ and tissue status and cochlear implant, malignant neoplasm, radiation therapy, HIV infection, and other hematological disease sickle-cell disorders

d Include Waterhouse-Friderichsen syndrome covering adrenal hemorrhage, anoxic brain damage, stroke

The IMD-related index hospitalization was associated with substantial mean and median costs of € 9,620 (SD € 22,197) and € 5,570 (IQR € 2,332), respectively (data not shown except mean cost in Fig 1). The results from the multivariable gamma regression model demonstrated that costs per day during the index hospitalization were 2.62 times and 3.37 times higher in patients with clinical presentation as meningitis plus sepsis (95% CI: 1.62–4.24) and sepsis only (95% CI: 2.19–5.20), respectively, than patients with meningitis only (Table 3). In addition, presence of risk factors according to STIKO was associated with 5.78 times higher of costs per day (95% CI: 3.26–10.25) compared to patients without risk factors. No statistically significant association was found for presence of IMD-related complications at discharge of the index hospitalization as well as for different age groups and sex.

**Table 3:** Adjusted cost ratios of total costs per day during IMD-related index hospitalization (2009–2015).

	Cost ratio^a^ (95%-CI)	p-value^a^
**Age (ref. <1 year)**		
1–4 years	1.04 (0.55–1.96)	0.906
5–9 years	2.08 (0.77–5.67)	0.150
10–17 years	0.95 (0.48–1.90)	0.890
18–24 years	1.10 (0.57–2.13)	0.773
> = 25 years	1.73 (0.91–3.27)	0.094
**Sex (ref. females)**	1.32 (0.91–1.92)	0.142
**Clinical presentation of IMD (ref. meningitis only)**		
Meningitis + sepsis	2.62 (1.62–4.24)	<0.001
Sepsis only	3.37 (2.19–5.20)	<0.001
Other IMD^b^	1.68 (0.93–3.03)	0.0828
**Presence of risk factor according to STIKO**^**c**^ **(ref. no)**^**c**^	5.78 (3.26–10.25)	<0.001
**Presence of IMD related complication at hospital discharge (ref. no)**^**d**^	1.27 (0.75–2.14)	0.370

CI = Confidence interval, IMD = Invasive meningococcal disease; STIKO = Standing Committee on Vaccination (Germany)

a obtained from multivariable gamma regression model

b Include Waterhouse-Friderichsen syndrome covering adrenal hemorrhage, meningococcal heart disease, other meningococcal infections and unspecified meningococcal disease

c Include functional or congenital asplenia, defects of the complement system, immunodeficiency with predominantly antibody defects, combined immunodeficiency, immunodeficiency associated with other major defects, common variable immunodeficiency, neutropenia and functional disorders of polymorphonuclear neutrophils, transplanted organ and tissue status and cochlear implant, malignant neoplasm, radiation therapy, HIV infection, and other hematological disease sickle-cell disorders

d Include Waterhouse-Friderichsen syndrome covering adrenal hemorrhage, anoxic brain damage, stroke

The results from the two-part regression model indicated that costs per day during the follow-up period were about 4 times higher in IMD patients older than 25 years (adjusted cost ratio (CR): 4.06, 95% CI 2.13–7.73) and about 2 times higher in patients aged 1–4 years (adjusted CR 2.09; 95% CI 1.03–4.25) and 10–17 years (adjusted CR: 2.16; 95% CI 1.04–4.49), respectively, compared to costs in patients aged <1 year (Table 4). Although the total cost per day were not statistically significant different with regard to sex and clinical presentation of IMD, the costs of patients with presence of risk factors according to STIKO (adjusted CR: 2.00; 95% CI: 1.19–3.36), or with presence of IMD-related complication (adjusted CR: 5.18; 95% CI: 1.31–20.50) or sequelae (adjusted CR: 2.34; 95% CI 1.52–3.59) during the follow-up period were statistically significant higher than that of those without.

**Table 4:** Adjusted cost ratios of total cost per day during the overall follow up period after discharge from IMD-related index hospitalization for all IMD patients.

	Cost ratio^a^ (95%-CI)	p-value^a^
**Age (ref. <1 year)**		
1–4 years	2.09 (1.03–4.25)	0.041
5–9 years	1.31 (0.42–4.04)	0.642
10–17 years	2.16 (1.04–4.49)	0.039
18–24 years	1.79 (0.87–3.70)	0.113
> = 25 years	4.06 (2.13–7.73)	<0.001
**Sex (ref. females)**	1.29 (0.88–1.90)	0.193
**Clinical presentation of IMD (ref. meningitis only)**		
Meningitis + sepsis	1.22 (0.71–2.19)	0.467
Sepsis only	1.22 (0.77–1.94)	0.392
Other IMD^b^	0.67 (0.35–1.29)	0.232
**Presence of risk factor according to STIKO**^**c**^ **(ref. no)**^**c**^	2.00 (1.19–3.36)	0.009
**Presence of IMD related complication at hospital discharge (ref. no)**^**d**^	0.40 (0.09–1.76)	0.224
**Presence of IMD related complication during follow-up (ref. no)**^**d**^	5.18 (1.31–20.50)	0.020
**Presence of IMD sequelae during follow-up (ref. no)**^**e**^	2.34 (1.52–3.59)	<0.001

CI = Confidence interval, IMD = Invasive meningococcal disease; STIKO = Standing Committee on Vaccination (Germany)

a obtained from two-part regression model composed of a logistic regression model in all patients with an indicator of non-zero costs as dependent variable, and a gamma regression model in patients with costs greater zero with costs as dependent variable

b Include Waterhouse-Friderichsen syndrome, meningococcal heart disease, other meningococcal infections and unspecified meningococcal disease

c Include functional or congenital asplenia, defects of the complement system, immunodeficiency with predominantly antibody defects, combined immunodeficiency, immunodeficiency associated with other major defects, common variable immunodeficiency, neutropenia and functional disorders of polymorphonuclear neutrophils, transplanted organ and tissue status and cochlear implant, malignant neoplasm, radiation therapy, HIV infection, and other hematological disease sickle-cell disorders

d Include Waterhouse-Friderichsen syndrome incl. adrenal hemorrhage, anoxic brain damage, stroke

e Include limb ataxia, paresis and paralysis, cranial nerve palsy, learning disbabilities and mental retardation, blindness, obstructive hydrocephalus, hearing loss, skin necrosis, limb amputation, chronic renal failure, epilepsy and seizures.

### Comparison of IMD cases with matched controls

For the comparison of HRU and costs between IMD cases and matched controls during the follow-up period, 164 IMD cases were matched to 656 controls without IMD. To improve the balance of HRU and costs in the baseline period, 17 (10%) IMD cases and 89 (14%) matched controls with extremely high baseline costs (defined as 75th percentile + 5*inter-quartile range of costs across all IMD and matched controls) were excluded resulting in a study population of 147 IMD cases and 567 matched controls. As a result of the matching, age categories and sex as well as the mean baseline costs were well balanced with slightly higher costs for all cost categories in the control group except for remedies and aids (S2 Appendix). The mean duration of follow-up was similar in IMD cases and matched controls with 48.3 months (SD 26.6 months)) and 49.9 months (SD 24.7 months), respectively (S2 Appendix). The distributions of number of matched IMD cases and controls within each study follow-up time were similar and were consistent with the distribution observed based on all IMD cases (S2 Appendix).

Overall, IMD cases had a significant higher rate of hospitalizations during the overall follow-up period than the matched controls (rate ratio (RR): 1.67; 95% CI 1.18–2.35). Although the rates of physician contacts in ambulatory care and different drug used were higher in IMD cases than controls, they were not statistically significant different (Table 5).

**Table 5:** Rate ratios of healthcare resource utilization during the study follow-up period (IMD cases vs. matched controls, 2009–2015).

	Hospitalizations		Physician contacts in ambulatory care		Different drugs used	
	Rate ratio (95% CI)^a^	p-value^a^	Rate ratio (95% CI)^a^	p-value^a^	Rate ratio (95% CI)^a^	p-value^a^
**Overall (up to 7 years)**	1.67 (1.18–2.35)	0.004	1.12 (0.99–1.26)	0.084	1.17 (1.00–1.36)	0.057
**0 month—<1 month**	26.89 (7.59–95.32)	<0.001	2.20 (1.79–2.71)	<0.001	2.04 (1.46–2.85)	<0.001
**1 month—<1 year**	1.76 (1.07–2.89)	0.026	1.24 (1.07–1.43)	0.004	1.26 (1.03–1.55)	0.024
**1 year—< 3 years**	1.19 (0.71–1.99)	0.505	0.97 (0.84–1.12)	0.669	1.02 (0.85–1.23)	0.825
**3 years—< 5 years**	1.03 (0.49–2.17)	0.934	1.05 (0.89–1.24)	0.579	0.91 (0.71–1.16)	0.448
**5 years—7 years**	2.58 (1.04–6.42)	0.041	1.06 (0.84–1.33)	0.641	0.89 (0.64–1.24)	0.480

^a^ obtained from negative-binomial regression models for each follow-up period

During the short-term follow-up period (i.e., < 1 year), rates of all HRU components were statistically significant higher in IMD cases compared to controls. The rate ratio was particularly high for hospitalization within 1 month after discharge from the IMD-related index hospitalization (RR: 26.89; 95% CI 7.59–95.32). During the long-term follow-up period (i.e., 1 year– 7 year), except hospitalizations during 5 to 7 years of the follow-up period (RR: 2.58; 95% CI 1.04–6.42), all other HRU components were not statistically significant different between IMD cases and matched controls.

**Fig 2:**
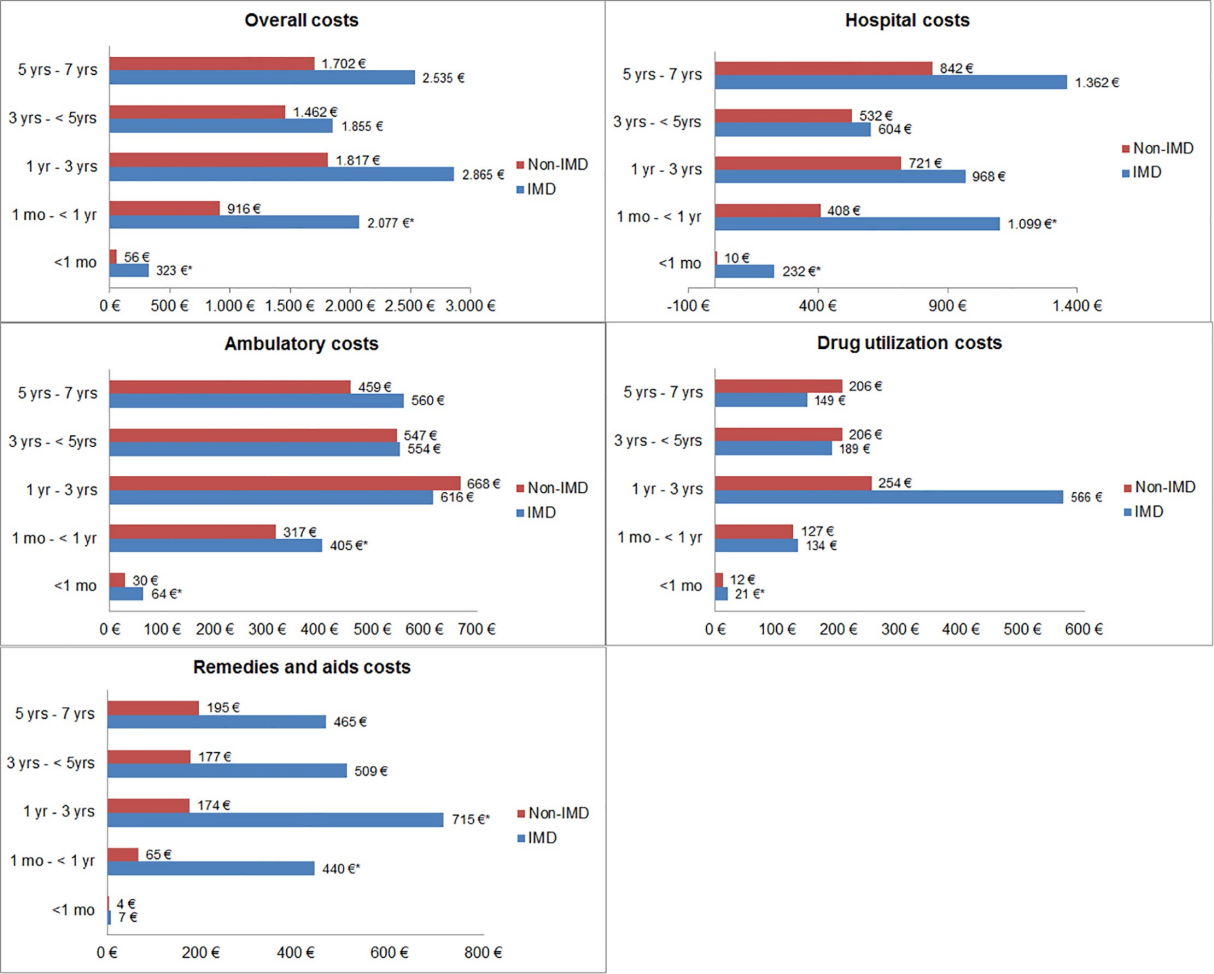
Mean costs (in Euro = €) of IMD cases and matched controls without IMD within pre-defined follow-up periods after IMD diagnosis, overall and stratified by healthcare sector. * statistically significant at bootstrap p-value <0.05 obtained from two-part regression model composed of a logistic regression model in all patients with an indicator of non-zero costs as dependent variable, and a gamma regression model in patients with costs greater zero with costs as dependent variable; IMD = Invasive meningococcal disease; based on data from n = 147 IMD cases and n = 567 matched controls without IMD.

For the comparison of short-term costs associated with HRU, the mean overall costs within the first month and within month 1 to year 1 of the follow-up period were statistically significant higher in IMD cases compared to matched controls (adjusted CRs: 6.02 and 2.32; 95% CIs: 2.53–11.02 and 1.22–3.87, respectively) (Table 6). These were mainly triggered by elevated hospital costs (Fig 2 and S2 Appendix). For the comparison of long-term costs associated with HRU, mean overall costs, hospital costs as well as remedies and aids costs were descriptively higher in IMD cases compared to controls; however, they were not statistically significant different except for the cost of remedies and aids in the follow-up period of 1 year to 3 years (Adjusted CR: 3.81; 95% CI: 1.04–9.03).

**Table 6:** Adjusted cost ratios of healthcare resource utilization during the study follow-up period (IMD cases vs. matched controls, 2009–2015).

	Overall Cost		Hospital Cost		Ambulatory Care Cost		Drug Cost		Remedies and Aids Cost	
	Rate ratio (Bootstrap 95% CI)^a^	Bootstrap p-value^a^	Rate ratio (Bootstrap 95% CI)^a^	Bootstrap p-value^a^	Rate ratio (Bootstrap 95% CI)^a^	Bootstrap p-value^a^	Rate ratio (Bootstrap 95% CI)^a^	Bootstrap p-value^a^	Rate ratio (Bootstrap 95% CI)^a^	Bootstrap p-value^a^
**Overall (up to 7 years)**	1.86 (1.14–2.80)	0.008	2.31 (1.06–4.37)	0.025	1.24 (0.93–1.71)	0.174	1.27 (0.73–2.15)	0.392	2.84 (1.02–6.06)	0.026
**0 month—<1 month**	6.02 (2.53–11.02)	0.001	24.23 (4.27–255.44)	0.005	2.32 (1.62–3.23)	0.001	1.80 (1.09–2.95)	0.023	1.81 (0.24–4.79)	0.354
**1 month—<1 year**	2.32 (1.22–3.87)	0.010	2.81 (1.15–6.00)	0.021	1.28 (1.08–1.51)	0.006	1.07 (0.71–1.61)	0.733	6.74 (1.10–20.22)	0.019
**1 year—< 3 years**	1.43 (0.85–2.23)	0.155	1.23 (0.61–2.33)	0.542	0.88 (0.72–1.05)	0.174	1.88 (0.60–4.46)	0.312	3.81 (1.04–9.03)	0.024
**3 years—< 5 years**	1.24 (0.77–1.90)	0.334	1.16 (0.42–2.45)	0.754	0.99 (0.75–1.28)	0.945	1.16 (0.40–2.68)	0.754	2.37 (0.31–5.52)	0.163
**5 years—7 years**	3.34 (0.71–8.45)	0.088	5.31 (0.41–17.71)	0.145	1.23 (0.80–1.83)	0.314	0.72 (0.32–1.37)	0.365	2.84 (0.62–6.37)	0.07

^a^ obtained from 2-part bootstrapping regression models for each follow-up period

## Discussion

In this study, we examined the demographic and clinical characteristics as well as healthcare resource use and related costs in 164 IMD patients selected from a source population of more than 8 million persons between 2009 and 2015 in Germany.

In general, IMD can occur in all age groups and mainly affects healthy individuals [22]. Nevertheless, IMD is facilitated by nonspecific damage of the mucosal barriers, e.g. by viral infections, dry air or smoking, as well as in individuals with underlying diseases such as immunodeficiency or congenital complement deficiencies [23,24]. Surprisingly, in our study only 13% had a documented risk factor according to STIKO recommendations for vaccination [25]. While this low percentage may be related to high vaccination coverage in “at-risk” populations and some IMD risk factors according to STIKO, e.g. persons at occupational risk or to traveling to countries with epidemic IMD spread, are not captured in claims data, it also indicates that important risk factors may be missing. In addition, it highlights that IMD is also frequent in subjects without such risk conditions.

The mean duration of the IMD-related index hospitalization observed in our study was slightly longer (13 days) compared with studies from the United States, Israel and the Netherlands ranging between 8.8 and 10 days [22,26,27]. From the multivariable analysis, we found that the length of hospital stay was longer in patients aged 25 years and older as well as in patients with clinical presentation as other IMD including patients with unspecified and possibly milder meningococcal infections that may have led to shorter hospitalizations. In contrast, another study from the United States reported a mean length of hospital stay of 17.8 days; however, the proportion of complicated IMD cases and the mean age was higher with 41% and 33.2 years, respectively [8]. In our study, we also showed that IMD-related complications observed during the IMD-related index hospitalization are associated with 51% increase of the length of hospital stay.

With regard to the clinical presentation of IMD, a good overall agreement with data of the national disease surveillance in Germany was observed with a slightly lower proportion of IMD patients with meningitis only (37.6% vs. 45.9%) and a similar proportion with sepsis (with or without meningitis) (51.3% vs. 53.8%) [28]. In previous studies from several Western countries, the distribution of clinical manifestations varied [7,14,22,26,27,29]. The reasons of these variations are not clear but may be explained by regional differences in serogroup distributions, diverging characteristics of study populations and differences in clinical practice, e.g. time of IMD diagnosis or diagnostic tests performed.

The 30-day and one-year mortality (4.3% and 5.5%, respectively) in our study was slightly lower than the mortality reported on the German national level between 2012 and 2015 with 9.6%, but similar to the year 2016 with six percent [30]. In general, our results are based on a low number of events and must be interpreted cautiously due to the low precision of estimates. The mortality reported from other countries were in a similar range between 6.4% and 11.6% [22,26,27,29].

In our study, we found that approximately 13% of IMD patients had an IMD-related complication at discharge of the IMD-related index hospitalization and 23.5% developed a sequela during the follow-up period. The number of IMD patients with sequelae observed in our study was lower compared to other studies ranging between 29% and 58% [7,8,14,22,26,27]; however, these studies are not directly comparable due to regional differences, different study populations (e.g. regarding age), different follow-up durations, diverging case definitions of IMD-related complications and sequelae and different data sources. For instance, Strifler et al. [31] conducted a systematic review on health outcomes in IMD cases and found that the most frequent sequelae attributable to IMD were hearing impairment, cognitive impairment and psychological problems; however, especially cognitive impairment and psychological problems are difficult to assess based on electronic healthcare databases [32] which may have led to an underestimation in our study. The most frequent complication was Waterhouse-Friderichsen syndrome covering adrenal hemorrhage (11.6%) and the most frequent sequelae was chronic renal failure (7.5%). In most other studies that investigated specific outcomes, renal complications and sequelae were not investigated or no events were reported [7,14,22,27,29,31]. Davis et al. [8] investigated the incidence of IMD complications within one year after initial diagnosis based on administrative claims data between 1998 and 2009 in the United States. Similar to our study, 9% of IMD cases experienced chronic renal failure but no events of adrenal haemorrhage were observed. However, Waterhouse-Friderichsen syndrome defined in our study covers but does not necessarily imply adrenal hemorrhage and cannot be directly compared with this study.

Further, our study indicates that IMD is associated with substantial costs, especially during the IMD-related hospitalization and within one year after discharged from hospital. In this context, the costs of the IMD-related index hospitalization costs were more than two-fold higher in patients with sepsis compared to patients with meningitis only and nearly six-fold increase in patients with underlying risk factor according to STIKO, emphasizing the need for preventive measures in patients with underlying risk conditions. In addition, the difference of mean costs between IMD cases and matched non-IMD controls were € 267 in the first month and € 1,161 from one month to one year after the IMD-related index hospitalization.

Long-term costs within seven years after diagnosis were substantially higher for IMD cases with documented risk factor according to STIKO as well as for those with a diagnosis of an IMD-related complication or sequela. Although the comparison of long-term costs between the IMD cases and the controls were not statistically significant different, the overall trend indicates that IMD is also likely to be associated with increased long-term costs. In this context, Scholz et al. [33] estimated total life time costs of € 19.6 (€ 57,100 per IMD case) to € 58.8 million (€171,000 per IMD case) including direct and indirect costs from a societal perspective for a hypothetical cohort of 343 patients with IMD caused by serogroup B in Germany.

Similar to costs, healthcare resource use was increased in the first year after IMD diagnosis compared to the general SHI population, but the difference became smaller in later time periods. The higher rate of hospitalizations during 5 to 7 years observed in IMD patients might be associated with IMD-related sequelae.

To our knowledge, the short and long-term costs and healthcare resource use of IMD compared to a matched control group without IMD was not investigated in Germany so far. Although other studies cannot be directly compared to ours due to, e.g., diverging reimbursement practices in different countries, differences regarding study design, and IMD case definitions and study populations, most revealed substantial short-term excess costs associated with IMD as observed in our study. Christensen et al. [34] derived the costs of acute IMD episodes from the diagnosis related group system used for inpatient billing in Germany in 2013 and estimated costs of € 5,045 and € 4,798 per IMD case aged < 16 years and 16+ years, respectively. However, the actual mean hospital costs in our study were substantially higher with costs of € 9,620. A similar result was found in a study from Australia in which the average cost of the acute IMD related hospitalization was essentially higher compared to the costs per case mix-adjusted separations in public hospitals ($ 12,312 vs. $ 4,918) [14]. In a Danish study including 2,902 IMD cases between 1980 and 2009 the patient’s initial costs were also elevated [32]. Another recently published Danish study showed that the total of the average attributable societal costs among 6,303 incident patients during the period from 1980 to 2015 were highest in the first year after diagnosis, with costs equaling USD 18,920 per event and hospital admission costs accounted for 65% and production loss for 30% [35]. Over the first 5-year period after diagnosis with meningococcal disease, average actual costs per patient were higher than their matched controls [35]. O’Brien et al. [26] investigated the costs of hospitalizations due to meningococcal disease in the United States through 1999 and 2001 with high mean costs of $ 23,294. As in our study higher costs in patients with sepsis were observed compared to those without. In a study from France, Bénard et al. [36] calculated the lifetime costs of severe meningococcal disease per case from all payer perspectives based on two hypothetical patient scenarios. The lifetime costs ranged between € 768,875 and € 2,267,251 depending on anticipated complications and sequelae. Like our study, the highest costs were estimated for the first year after IMD diagnosis accounting for 8.3 to 21.7% of lifetime costs.

### Limitations

Although the analysis dataset obtained from the InGef database covered more than 8 million insured members of SHIs in Germany, representativeness for the whole German population cannot be guaranteed. Therefore, the demographic and clinical characteristics as well as other outcomes obtained in this study may not be generalizable to the whole German SHI population. However, the InGef database has shown good overall accordance with the population of Germany with regard to morbidity, mortality and drug usage [15].

Another limitation of this study is the low observed number of IMD cases. Despite the large analysis data set, only 164 incident IMD cases were eligible for analysis. Due to the rarity of the disease and data protection reasons, in-depth analyses (e.g. by age, clinical presentation of IMD at a regional level such as Federal State) could not be conducted. In addition, the statistical power to detect differences regarding costs and healthcare resource use in IMD cases and matched controls without IMD was low due to the low sample size. In this context, the p-values and confidence intervals of the regression analyses on costs must be interpreted with caution due to the assumed non-normal distribution of cost variables and the low sample size, especially in the two-part model on total costs per day during follow-up in which modelling of zero costs may lead to further instability of the estimators. In this case, we therefore calculated bootstrap confidence intervals and p-values, which do not require distributional assumptions about the parameter estimators.

As our study did not include a review of individual patient files to assess detailed clinical and laboratory criteria to confirm the presence of medical conditions, which for data protection reasons is generally not feasible, misclassification of IMD cases in general and with regard to clinical presentation, e.g., with or without sepsis, cannot be ruled out. We therefore only considered documented main discharge diagnoses in the hospital setting, since these diagnoses are used for reimbursement and are expected to have a higher coding quality.

As a further limitation of this study, it was not possible to differentiate between distinct serogroups of N. meningitidis, e.g. A, B, C, W and Y, causing IMD because this information cannot be derived from ICD-10 GM codes.

Regarding costs, only direct medical costs from the perspective of German SHIs were included in the analyses. Other or indirect costs associated with sick leave, rehabilitation, disability pension or absenteeism from work were not included due to lack of information.

## Conclusions

IMD resulted in severe complications and sequelae and was associated with extensive costs and increased healthcare resource use in Germany, especially in the first year after IMD diagnosis and due to the IMD-related hospitalization. In general, these data underline the importance of preventive measures against IMD, e.g. vaccinations against different serogroups, to further reduce the number of IMD cases and life-long impairment in children and adolescents as well as to avoid related costs from payer perspective. Additional studies with larger sample sizes of IMD cases are required to further investigate the long-term effects of IMD as well as underlying risk factors as possible indications for vaccinations.

## Supporting information

S1 Appendix Definition of risk factors for IMD, complications and sequelae. (DOCX)

S2 Appendix Costs and health resource use by clinical manifestation of IMD and in matched IMD cases and controls without IMD. (XLSX)
