# Supplementary material for: Clinical and economic burden of invasive meningococcal disease: Evidence from a large German claims database
Source: PLoS One. 2020 Jan 28;15(1):e0228020. doi: 10.1371/journal.pone.0228020 (PMC6986746; doi:10.1371/journal.pone.0228020)
Supplement: S1 Appendix — (DOCX) [file pone.0228020.s001.docx]

Table 1 Definition of risk factors for IMD, complications and sequelae

| **Variable** | **Description** | **Operational definition** |
| --- | --- | --- |
| Defects of the complement system | Immune deficiency or immune comprised therapy / risk factor according to STIKO | Patients with a hospital diagnosis or verified ambulatory diagnosis (ICD-10 GM code) D84 prior to the index date |
| Immunodeficiency with predominantly antibody defects | Immune deficiency or immune comprised therapy / risk factor according to STIKO | Patients with a hospital diagnosis or verified ambulatory diagnosis (ICD-10 GM code) D80 prior to the index date |
| Combined immunodeficiencies | Immune deficiency or immune comprised therapy / risk factor according to STIKO | Patients with a hospital diagnosis or verified ambulatory diagnosis (ICD-10 GM code) D81 prior to the index date |
| Immunodeficiency associated with other major defects | Immune deficiency or immune comprised therapy / risk factor according to STIKO | Patients with a hospital diagnosis or verified ambulatory diagnosis (ICD-10 GM code) D82 prior to the index date |
| Common variable immunodeficiency | Immune deficiency or immune comprised therapy / risk factor according to STIKO | Patients with a hospital diagnosis or verified ambulatory diagnosis (ICD-10 GM code) D83 prior to the index date |
| Neutropenia  and Functional disorders of polymorphonuclear neutrophils | Immune deficiency or immune comprised therapy / risk factor according to STIKO | Patients with a hospital diagnosis or verified ambulatory diagnosis (ICD-10 GM code) D70, D71 prior to the index date |
| Transplanted organ and tissue status and cochlear implant | Immune deficiency or immune comprised therapy / risk factor according to STIKO | Patients with a hospital diagnosis or verified ambulatory diagnosis (ICD-10 GM code) Z94, Z96.2 or an OPS-Code 52092, 52097, 52098 prior to the index date |
| Malignant neoplasm | Immune deficiency or immune comprised therapy / risk factor according to STIKO | Patients with a hospital diagnosis or verified ambulatory diagnosis (ICD-10 GM code) C00-C96 prior to the index date |
| Radiation therapy | Immune deficiency or immune comprised therapy / risk factor according to STIKO | Patients with a hospital diagnosis or verified ambulatory diagnosis (ICD-10 GM code) D90 prior to the index date |
| HIV infection | Risk factor according to STIKO | Patients with a hospital diagnosis or verified ambulatory diagnosis (ICD-10 GM code) B20-B24, Z21 prior to the index date |
| Other hematological diseases und sickle-cell disorders | Risk factor according to STIKO | Patients with a hospital diagnosis or verified ambulatory diagnosis (ICD-10 GM code) D55-D59, E83.1, D57 prior to the index date |
| Adrenal hemorrhage | IMD complication at hospital discharge and during follow-up | Patients with a hospital diagnosis (ICD-10 GM code) A39.1 at hospital discharge of the IMD related hospitalization or during follow-up |
| Anoxic brain damage | IMD complication at hospital discharge and during follow-up | Patients with a hospital diagnosis (ICD-10 GM code) G93.1 at hospital discharge of the IMD related hospitalization or a hospital diagnosis or verified ambulatory diagnosis during follow-up |
| Stroke | IMD complication at hospital discharge and during follow-up | Patients with a hospital diagnosis (ICD-10 GM code) I60-I64 during follow-up at hospital discharge of the IMD related hospitalization or during follow-up |
| Limb ataxia, paresis, and paralysis. | IMD sequelae | Patients with a hospital diagnosis or verified ambulatory diagnosis (ICD-10 GM code) G04.1, G11.1, G11.2, G81, G82, G83.0-G83.4, G83.9 or R27.0 during follow-up |
| Cranial nerve palsy | IMD sequelae | Patients with a hospital diagnosis or verified ambulatory diagnosis (ICD-10 GM code) G51.0, G53, H49.0-H49.2 during follow-up |
| Learning disabilities and mental retardation | IMD sequelae | Patients with a hospital diagnosis or verified ambulatory diagnosis (ICD-10 GM code) F70-F73, F78, F79, F81 during follow-up |
| Blindness | IMD sequelae | Patients with a hospital diagnosis or verified ambulatory diagnosis (ICD-10 GM code) H54.0, H54.4 during follow-up |
| Obstructive hydrocephalus | IMD sequelae | Patients with a hospital diagnosis (ICD-10 GM code) G91 during follow-up |
| Hearing loss | IMD sequelae | Patients with a hospital diagnosis or verified ambulatory diagnosis (ICD-10 GM code) H90, H91.2-H91.9 during follow-up |
| Skin necrosis and/or skin grafting | IMD sequelae | Patients with a hospital diagnosis L51, L88, L90.5, R02 or an OPS code 5901, 5902, 5904, 5905 (skin grafting) during follow-up |
| Limb amputation | IMD sequelae | Patients with an OPS code 5862*-5865* during follow-up |
| Chronic renal failure | IMD sequelae | Patients with a hospital diagnosis or verified ambulatory diagnosis I12.0, I13.1, I13.2, N01*, N03*, N04*, N05*, N18*, N19*, Z49x, Z94.0, Z99.2 or an OPS code 8853, 8854, 8855, 8857 (dialysis) or EBM code (13602, 13610, 13611, 40823-40828) (dialysis) during follow-up |
| Epilepsy and seizures | IMD sequelae | Patients with a hospital diagnosis or verified ambulatory diagnosis (ICD-10 GM code) G40, G41, R56.8 during follow-up |

EBM = Einheitlicher Bewertungsmaßstab (Doctors’ Fee Scale within the Statutory Health Insurance Scheme), ICD-10 GM = German Modification of the International Classification of diseases 10th revision, IMD = Invasive meningococcal disease, OPS Code = Operationen und Prozedurenschlüssel (German Procedure Classification), STIKO = German Standing Vaccination Committee
